# Patient-specific instrumentation does not improve tibial component coronal alignment for medial UKA compared to conventional instrumentation

**DOI:** 10.1186/s40634-020-00257-3

**Published:** 2020-06-08

**Authors:** Houssam Kalache, Jacobus H. Müller, Mo Saffarini, Evrard Gancel

**Affiliations:** 1 Centre Hospitalier Privé Saint-Grégoire, 6 Boulevard de la Boutière, 35760 Saint-Grégoire, France; 2 ReSurg S.A, Rue Saint-Jean 22, 1260 Nyon, Switzerland

**Keywords:** UKA, PSI, Conventional instrumentation, HKA angle, mMPTA

## Abstract

**Background:**

Patient-specific instrumentation (PSI) may potentially improve unicompartmental knee arthroplasty (UKA) implant positioning and alignment. The purpose of this study was to compare early radiographic coronal alignment of medial UKA performed using PSI versus conventional instrumentation (CI) for tibial resections.

**Methods:**

A consecutive series of 47 knees (47 patients) received medial UKA, with the tibial resections performed using CI (first 22 knees) or PSI (next 25 knees), while femoral resections were performed with CI in both groups. The target mechanical medial proximal tibial angle (mMPTA) was 87° ± 3°, and the target hip-knee-ankle (HKA) angle was 177° ± 2°. The postoperative mMPTA and HKA were evaluated from postoperative radiographs at a follow-up of 2 months.

**Results:**

Differences in postoperative mMPTA (*p* = 0.509) and HKA (*p* = 0.298) between the two groups were not statistically significant. For the mMPTA target, 24% of knees in the PSI group (85.6° ± 2.1°) and 32% of the CI group (85.0° ± 3.6°) were outliers. For the HKA target, 44% of knees in the PSI group (176.3° ± 2.8°) and 18% of the CI group (177.1° ± 2.3°) were outliers. Considering the two criteria simultaneously, 60% of knees in the PSI group and 45% of knees in the CI group were outside the target zone (*p* = 0.324), whereas 28% of knees in the PSI group and 41% of knees in the CI group were outside the target zone by more than 1° (*p* = 0.357).

**Conclusions:**

The results of the present study revealed no statistically significant difference in radiographic coronal alignment of UKA performed using PSI versus CI for tibial resections.

## Background

Unicompartmental knee arthroplasty (UKA) was introduced by Marmor [1] in the 1970s as a less invasive treatment than total knee arthroplasty (TKA) for unicompartmental tibiofemoral arthritis. Its main benefits are bone-sparing cuts and preservation of the cruciate ligaments [2], which enable restoration of close-to-normal native biomechanics [3], particularly for knees with medial compartment arthritis [4, 5].

Advances in prosthesis design, surgical techniques and patient selection have led to improved outcomes and survival of UKA [6], but national registries still indicate inferior long-term survival compared to TKA [7, 8], mainly due to the progression of arthritis to adjacent compartments [9–11]. Several authors observed prosthetic malalignment to be a principal risk factor for the progression of arthritis and early failure [10, 12–14]. It is worth noting, however, that UKA revision rates tend to decrease with surgeon volume or experience [15], hence the recommendations that surgeons offering UKA should perform an annual minimum of 11 to 23 knees [7, 16, 17].

Patient-specific instrumentation (PSI) was introduced to improve implant positioning and alignment [13, 18], notably to help less experienced surgeons achieve improved clinical and radiographic outcomes [17]. While some studies revealed that PSI improves accuracy of alignment for both TKA [19, 20] and UKA [21, 22], the differences are often insignificant [23]. Conversely, a recent randomized controlled trial reported decreased accuracy in UKA tibial alignment when using patient-specific tibial cutting guides, with deeper tibial resections, compared to conventional instrumentation [24]. Therefore, uncertainty remains on whether PSI yields more accurate coronal alignment compared to conventional instrumentation (CI). The purpose of this study was therefore to compare early radiographic coronal alignment of UKA performed using PSI versus CI for tibial resections. The hypothesis was that both types of instrumentation would render equivalent early radiographic coronal alignment.

## Material and methods

### Patients

A consecutive series of 47 knees (47 patients) received medial UKA using either CI or PSI to perform tibial resections over two consecutive years by the senior surgeon (EG) at the same center. Indications for surgery were localized medial tibiofemoral osteoarthritis (Ahlbäck grade ≥ 2), with medial tibiofemoral pain, partial or complete intra-articular varus deformity < 10°, and extension deficit ≤5°. Tibial resections were performed using CI in the first 22 knees and PSI in the next 25 knees.

### Patient consent

All patients provided informed consent for the participation in the study and the use of their data and images for research purposes.

### Preoperative planning

**Fig. 1:**
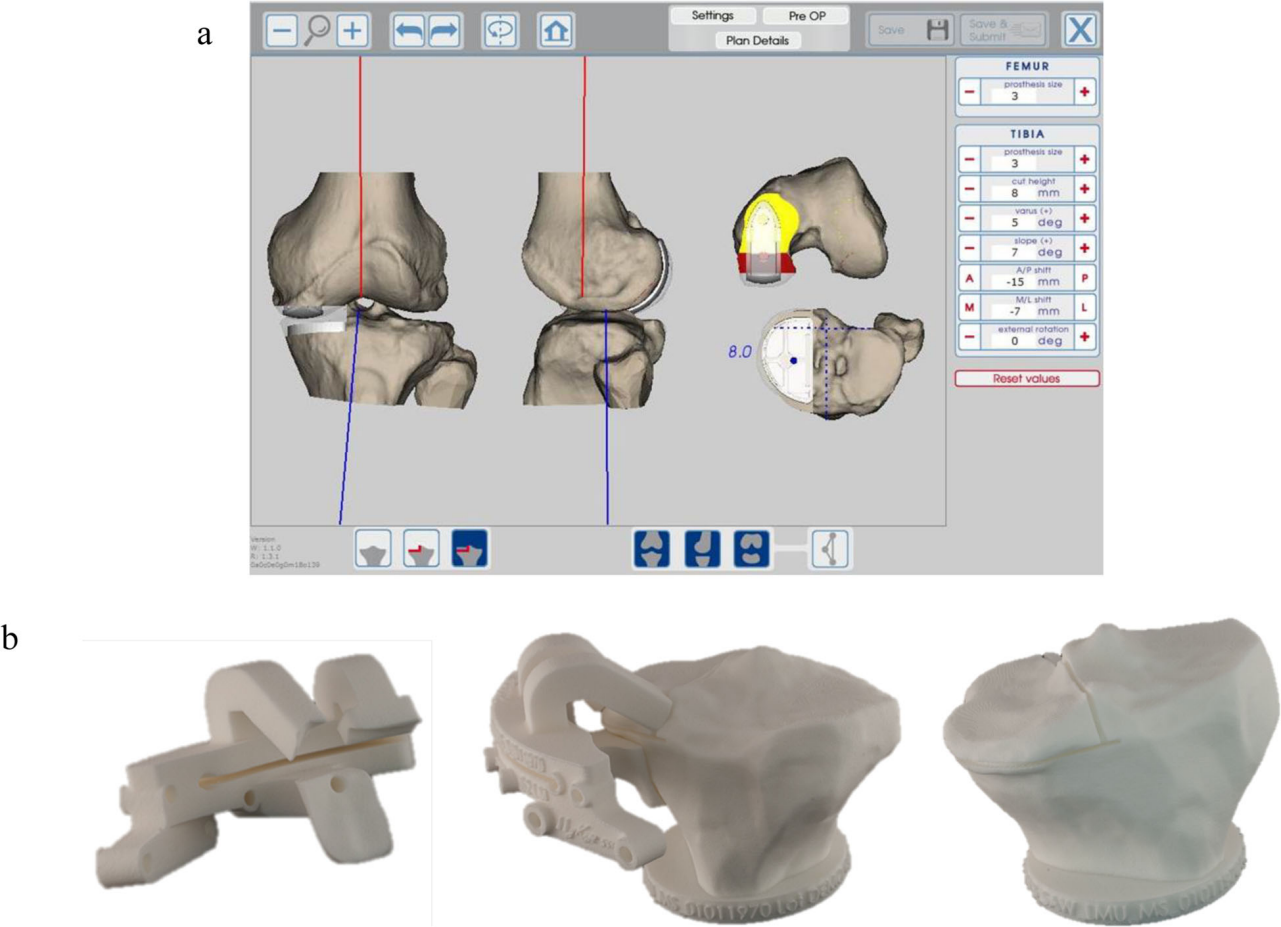
Stages of the preoperative planning workflow. a) Design of the PSI guide. b) Verification of the PSI guide on 3Dprinted models

Patients scheduled for UKA using PSI for the tibial resections had a preoperative Computed Tomography (CT) scan of the affected knee, with additional cross sections through the hip and ankle to determine the Hip-Knee-Ankle (HKA) angle. The tibial resection PSI guides were based on the surgical guidelines which aimed for a tibial resection of 6 mm, mechanical medial proximal tibial angle (mMPTA) of 87°±3° and HKA angle of 177° ± 2° (Fig. 1) [10]. The tolerance of up to 3° deviation was allowed to leave some residual varus in cases with greater deformity [2].

### Surgical technique

**Fig. 2:**
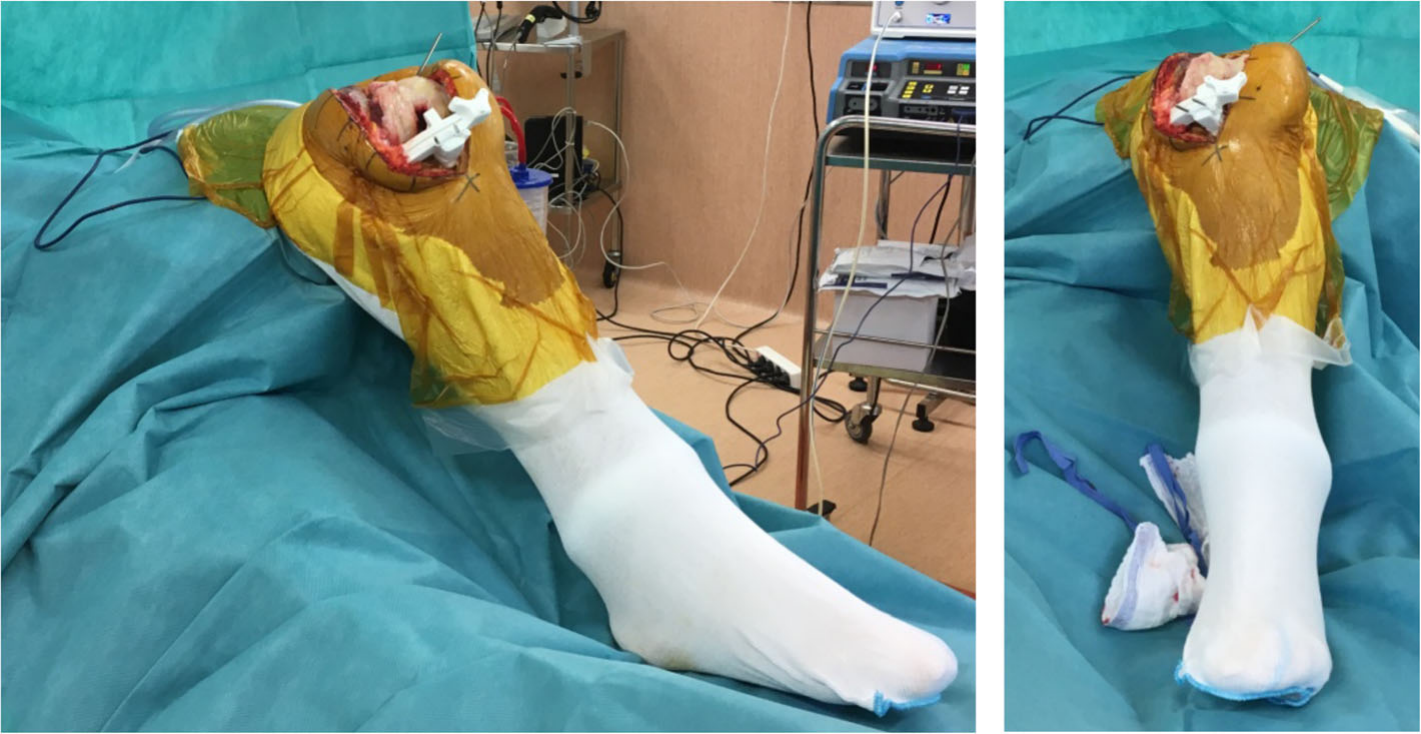
Fixation of the tibial PSI cutting guide

All patients received a fixed bearing UKA (MyKnee UNI#, Medacta, Castel San Pietro, Switzerland) following a standardized surgical technique with a pneumatic tourniquet. A minimally invasive medial parapatellar incision was made extending from the superior pole of the patella to 3 cm below the joint space. The PSI cutting guide was fixed with three pins to the proximal tibia, with the resection level measured from the deepest point on the medial tibial plateau (Fig. 2). Using an oscillating saw, the sagittal cut was performed first, followed by the axial cut, before the tibial insert size was validated. The resection level was systematically measured to ensure consistency with the preoperative plan of 6 mm. In the CI group, the tibial cut was performed using an extramedullary guide, aiming for a resection level of 4 or 6 mm (depending on the amount of cartilage wear), mMPTA of 87° ± 3° and HKA angle of 177° ± 2°.

In both groups, the distal and posterior femoral resections were performed next by using a dependent-cut guide to determine the distance from the tibial cut. The aim was to achieve an HKA angle target of 175° to 179°, and a varus-valgus laxity of 1 to 2 mm both in flexion and in extension. Alignment was verified for both groups using an extramedullary guide. After verification of component sizes with trials, the UKA components were cemented in place (Palacos R + G, Zimmer, Wehrheim, Germany). Tourniquet time was recorded for both groups.

### Radiographic assessment

For both groups, frontal, sagittal and HKA radiographs were obtained preoperatively and at 2 months follow-up to measure the HKA angle and mMPTA.

### Statistical analysis

Descriptive statistics were used to summarize the data and Shapiro-Wilk tests were used to assess the normality of distribution. For non-Gaussian quantitative data, differences between groups were evaluated using the Wilcoxon rank sum test (Mann Whitney U test), and a Fisher’s exact test was used to compare discrete quantitative variables. Considering the findings of Kerens et al. [25] who reported mMPTA to have a standard deviation of 3.6°, the required number of subjects was 44 (22 in each group) to achieve a power of 80% at a 5% level of significance. Statistical analyses were performed using R version 3.4.2. (R Foundation for Statistical Computing, Vienna, Austria). A *p*-value < 0.05 was considered statistically significant.

## Results

The mean age, BMI and tourniquet time were similar between the two groups, but there were significantly (*p* = 0.002) less women in the PSI group (*n* = 6 (28%)) compared with the CI group (*n* = 15 (68%)) (Table 1).

**Table 1:** Comparison of UKA using PSI versus using conventional instrumentation for tibial resections

	PSI (*n* = 25)		Conventional (*n* = 22)		
	mean ± SD n (%)	(min–max)	mean ± SD n (%)	(min–max)	*p value*
Patient demographics					
Age (years)	65.1 ± 9.2	(46–86)	70.5 ± 9.2	(52–85)	0.050
BMI (kg/m^2^)	27.3 ± 2.9	(21.3–32.2)	26.4 ± 3.6	(21–33)	0.365
Women	6 (24%)		15 (68%)		**0.002**
Right knee	15 (60%)		12 (55%)		0.709
Intraoperative measurements					
Tourniquet time (min)	58 ± 6	(45–75)	58 ± 9	(50–90)	0.55
Coronal alignment					
HKA angle (deg)					
Preoperative	173.8 ± 2.9	(167–178)	173.6 ± 3.0	(169–181)	0.844
Postoperative	176.3 ± 2.8	(170–183)	177.1 ± 2.3	(171–181)	0.298
*Net change*	*2.5 ± 1.5*	*(−1–5)*	*3.4 ± 2.4*	*(−4–6)*	*0.114*
mMPTA (deg)					
Preoperative	83.1 ± 2.0	(80–87)	84.7 ± 2.0	(81–88)	**0.012**
Postoperative	85.6 ± 2.1	(80–89)	85.0 ± 3.6	(78–92)	0.509
*Net change*	*2.4 ± 2.6*	*(−2–7)*	*0.3 ± 4.1*	*(−7–7)*	***0.045***

*Abbreviations*: *PSI* patient specific instruments, *BMI* body mass index, *HKA* hip-knee-ankle, *mMPTA* mechanical medial proximal tibial angle, *min* minutes, *deg* degrees

Preoperative mMPTA was significantly (*p* < 0.012) smaller in the PSI group (83.1° ± 2.0°) compared with the CI group (84.7° ± 2.0°), but the postoperative mMPTA was similar between the two groups (Table 1). It is worth noting that the net change in mMPTA was significantly (*p* = 0.045) greater in the PSI group (2.4° ± 2.6°) compared with the CI group (0.3° ± 4.1°). The pre- and post-operative HKA angles were similar between the two groups.

**Fig. 3:**
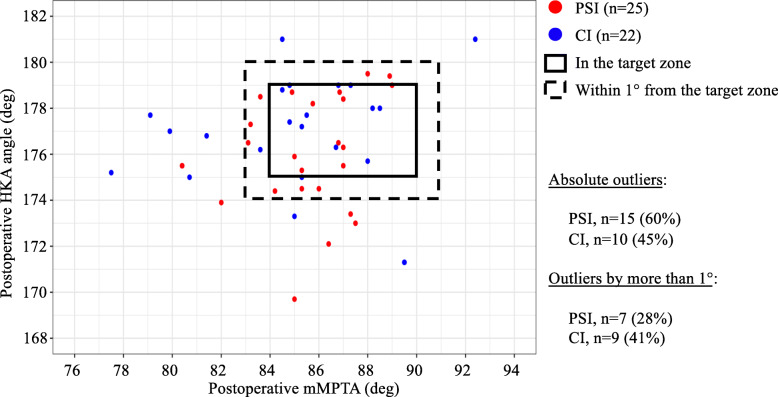
Comparison of UKA using PSI versus conventional instrumentation for tibial component alignment to achieve the coronal alignment target zone

Considering the criteria for coronal alignment of mMPTA between 87° ± 3°, 24% of knees in the PSI group and 32% of the CI group were strictly outliers (*p* = 0.554), while only 8% of knees in the PSI group and 27% of the CI group were outliers by more than 1° (*p* = 0.083). Considering the criteria for HKA angle between 177° ± 2°, 44% of knees in the PSI group and 18% of the CI group were strictly outliers (*p* = 0.061), while only 24% of knees in the PSI group and 18% of the CI group were outliers by more than 1° (*p* = 0.630). Considering the two criteria simultaneously (Fig. 3), 60% of knees in the PSI group and 45% of knees in the CI group were strictly outside the target zone (*p* = 0.324), while 28% of knees in the PSI group and 41% of knees in the CI group were outside the target zone by more than 1° (*p* = 0.357). The differences in proportions of outliers were not statistically significant between the two groups.

## Discussion

The main finding of this study was that the difference in radiographic coronal alignment of UKA performed using PSI versus CI for tibial resections is not statistically significant. This finding therefore supports the hypothesis that PSI does not improve tibial component coronal alignment for UKA compared to CI. The clinical relevance of the findings was that, compared to CI, PSI yielded less outliers from the target mMPTA but more outliers from the target HKA angle, although the differences were not statistically significant. When the two criteria were considered simultaneously, the proportion of absolute outliers was greater in the PSI group, but the proportion of outliers by more than 1° was greater in the CI group.

Previous studies [13, 18] have demonstrated superior accuracy for implant positioning in UKA using PSI compared to CI. In the present study, accuracy in coronal alignment of UKA was equivalent using PSI versus CI. This conflicting finding might be due to the experience level of the senior surgeon, who performs 40 UKAs annually, which is considerably greater than the range of 11 to 23 annually, as suggested by guidelines [1, 7, 16]. Moreover, Sanz-Ruiz et al. [17] highlighted the true value of PSI to be improvement of clinical and radiographic outcomes of UKA by inexperienced surgeons, during their learning curve.

The reported radiographic outcomes for mMPTA and HKA angle of the present study compare well to previous findings. In a prospective study, Kerens et al. [25] compared 30 UKAs using PSI to 30 UKAs using CI. The mMPTA was 89° (range, 83° to 93°) using PSI and 88° (range, 80° to 95°) using CI, and HKA angle was 176° (range, 168° to 182°) using PSI and 176° (range, 169° to 182°) using CI. In randomized controlled trial, Ollivier et al. [26] compared 30 UKAs using PSI to 30 UKAs using CI. The mMPTA was 89° (range, 88° to 93°) using PSI and 89° (range, 87° to 92°) using CI, and HKA angle was 178° (range, 176°–182°) using PSI and 178° (range, 175° to 182°) using CI. Leenders et al. [27] published results of a continuous series of 129 UKAs using PSI, and reported mMPTA of 90.9° (range, 81.4° to 99.6°) and HKA angle of 176.4° ± 3.4°. In the present study, there were considerable proportions of outliers in both the PSI and CI group, though comparison to proportions reported in the literature is difficult due to differences in definitions of target zones.

Malpositioning of the tibial component in UKA increases the risk of component migration and loosening, leading to higher revision rates, especially in low volume surgical centres [28]. Assistive technologies like PSI and robotic surgery has the potential to improve component positioning, but it is unclear which technology is best suited to this task [29]. Recent findings suggest that, compared to UKA using CI, robot-assisted UKA grants improved restitution of the joint-line height, leads to lower alignment outliers and revision rates [30, 31]. Interestingly, a recent randomised control trial [29] revealed that PSI for tibial component positioning in UKA resulted in comparable accuracy to a robotic system. However, the use of PSI does not guarantee accurate tibial rotational alignment [32] and may lead to increased operating time, reduced accuracy and considerable costs [33]. The findings of the present study revealed comparable coronal alignment between PSI and CI, and therefore does not support the routine use of PSI for medial UKA.

These findings of the present study need to be interpreted with the following limitations in mind. First, this was a retrospective study without any randomization. However, the radiographs were systematically obtained as part of normal follow-up for UKA, and blinded before measurement of the radiographic outcomes. Second, preoperative mMPTA was statistically different between the two groups, but it was by random chance since CI was used on the first 22 knees and PSI on the next 25 knees. Moreover, although statistically significant, a mean difference of 1.6° is not clinically relevant. Third, no clinical scores were recorded, and it is unclear if the radiographic outcomes are related to clinical outcomes. Fourth, the study might be underpowered due to the relatively small number of knees in the two groups. Fifth, radiographic measurements were obtained at 2 months follow-up, and some knees could still have residual stiffness with some amount of flexion contracture. This might affect the radiographic analysis and alter the measurements, although all patients followed the same rehabilitation protocol.

## Conclusion

The results of the present study revealed no statistically significant difference in radiographic coronal alignment of UKA performed using PSI versus CI for tibial resections. In UKA performed by a high-volume surgeon, PSI did not improve tibial component alignment compared to CI. The choice of PSI versus CI requires careful consideration, especially for experienced surgeons, since the present study could not demonstrate any radiographic benefit.

## Data Availability

Not applicable.

## Abbreviations

PSI: Patient-specific instrumentation
UKA: Unicompartmental knee arthroplasty
CI: Conventional instrumentation
mMPTA: Mechanical medial proximal tibial angle
HKA: Hip-knee-ankle
TKA: Total knee arthroplasty
CT: Computed tomography
